# Competitive lottery-based assembly of selected clades in the human gut microbiome

**DOI:** 10.1186/s40168-018-0571-8

**Published:** 2018-10-19

**Authors:** Adrian J. Verster, Elhanan Borenstein

**Affiliations:** 1 Department of Genome Sciences, University of Washington, Seattle, WA 98195 USA; 2 Blavatnik School of Computer Science, Tel Aviv University, 6997801 Tel Aviv, Israel; 3 Sackler Faculty of Medicine, Tel Aviv University, 6997801 Tel Aviv, Israel; 4 Department of Computer Science and Engineering, University of Washington, Seattle, WA 98195 USA; 5 Santa Fe Institute, Santa Fe, NM 87501 USA

**Keywords:** Ecology, Competitive lottery model, Microbiome assembly, Computational modeling, Human gut microbiome

## Abstract

**Background:**

While the composition of the gut microbiome has now been well described by several large-scale studies, models that can account for the range of microbiome compositions that have been observed are still lacking. One model that has been well studied in macro communities and that could be useful for understanding microbiome assembly is the competitive lottery model. This model posits that groups of organisms from a regional pool of species are able to colonize the same niche and that the first species to arrive will take over the entire niche, excluding other group members.

**Results:**

Here, we examined whether this model also plays a role in the assembly of the human gut microbiome, defining measures to identify groups of organisms whose distribution across samples conforms to the competitive lottery schema. Applying this model to multiple datasets with thousands of human gut microbiome samples, we identified several taxonomic groups that exhibit a lottery-like distribution, including the *Akkermansia*, *Dialister*, and *Phascolarctobacterium* genera. We validated that these groups exhibit lottery-like assembly in multiple independent microbiome datasets confirming that this assembly schema is universal and not cohort specific. Examining the distribution of species from these groups in the gut microbiome of developing infants, we found that the initial lottery winner can be replaced by a different member of the group. We further found that species from lottery-like groups tend to have fewer genes in their genomes, suggesting more specialized species that are less able to engage in niche differentiation.

**Conclusions:**

Combined, our findings highlight the complex and dynamic process through which microbial communities assemble and suggest that different phylogenetic groups may follow different models during this process.

**Electronic supplementary material:**

The online version of this article (10.1186/s40168-018-0571-8) contains supplementary material, which is available to authorized users.

## Background

The human gut microbiome is a complex ecosystem that harbors hundreds of bacterial taxa and is tightly linked to our health [1, 2]. Efforts to characterize the composition of the microbiome, using either marker gene-based approaches or shotgun metagenomics, have found not only compositional shifts associated with host disease, but also tremendous variation across healthy individuals [1, 3]. Indeed, early attempts to identify a core microbiome—a set of species that are shared between all healthy hosts—were generally unsuccessful, suggesting that such shared species comprise only a small fraction of the overall microbiome composition [4–6].

In an attempt to characterize patterns of such microbiome variation, many studies have focused on inferring specific relationships between species, viewing the assembly of the microbiome as an outcome of such interactions [7]. Multiple studies, for example, have set out to identify species pairs that tend to co-occur across samples and developed methods for characterizing the network of such co-occurring species in the microbiome [8–11]. Other studies have attempted to partition microbiome species into clusters of co-occurring species. For example, a study of the composition of the gut microbiome post antibiotic treatment identified two groups of species: those that are sensitive to the antibiotic treatment and those that are resistant [12]. Similar attempts in non-host-associated communities have also found clusters of co-occurring species in each stage of the developing apple flower microbiome [13] and clusters of species which have similar seasonal variations in abundance in the microbiome of a lake [14]. Notably, while such studies often do not explicitly define the mechanistic interpretation of a co-occurring group, such groups could be thought of as ecological guilds [15], representing, for example groups of organisms that perform a similar function within the ecosystem (e.g., different steps of the nitrogen cycle [16] or bioreactor degradation [17]) or that have overlapping nutrient requirements and are co-filtered by niche selection [18, 19].

Importantly, however, this “deterministic” assembly model, where guilds of functionally similar species are being selected by the environment, may not account for the extreme variation observed in microbial communities across seemingly similar environments. An alternative perspective puts more emphasis on stochastic effects in community assembly [20, 21]. For example, when *Caenorhabditis elegans* is colonized by two selectively neutral bacterial strains that differ only in the presence of a marker gene, the gut community is ultimately dominated by one or the other, suggesting that stochastic forces govern the assembly of this community [22]. Moreover, recent evidence suggests that there is in fact a balance between niche and stochastic factors in community assembly in the microbiome [23]. One approach that has been suggested to combine these factors is the notion of priority effects, which states that the final community assembly is often governed by the order at which species arrive during colonization. For example, the species that arrives to the community first can become entrenched, preventing other species with a similar niche from joining the ecosystem. Such priority effects have been characterized extensively in macroecology [24, 25] and have been shown to also govern the assembly of microorganisms in flower nectar communities [26], as well as the colonization of *Bacteroides* species in the mouse gut [27].

Another promising approach for combining niche and stochastic factors in community assembly is the competitive lottery model [28]. This model posits a competition within a well-defined pool of potential colonizing species for a given niche space and that the niche can support only a single species from this pool (as in a strong priority effect). This model further assumes that the “winning” species is determined randomly (hence the name “lottery”) owing to various stochastic processes and, accordingly, that different geographical locations will have different lottery winners independent of any niche effects. This model was originally proposed to explain the ecology of reef fishes [28], in which the lottery winner occupies a specific patch on the reef and excludes other fish from that patch. Each newly opened patch will be similarly filled by a single fish (determined randomly as the first to arrive) and once occupied will not be displaced due to strong priority effects. Since different patches are occupied by different lottery winners from different species, this model may account for the coexistence of competing species across the entire reef. Beyond reef fishes, this model has been extended to flowering plants [29], parasites [30], and the microbiome of the algae *Ulva australis* [31]. In the last case, for example, it has been shown that the microbiome is distinct from the surrounding seawater, implying selection for specific niches on the algae surface, but that it also varies tremendously between communities, suggesting that stochastic forces determine the specific species that dominate each community [32]. The researchers postulated that there were functionally equivalent groups of bacteria and that from each such group a single member colonized *U. australis* and excluded the rest of the group.

To date, however, there has not been any effort to systematically test the extent to which the competitive lottery schema applies to the human gut microbiome or to identify groups of species in this microbiome that may follow this schema. The human gut microbiome represents a well-defined microbial community, harboring a few hundred strains [2], and most of its members have been fully sequenced. In analogy to the reef fish ecosystem, a lottery-governed group of microbial species in the human gut microbiome would account both for specific species compositional patterns in the microbiome (e.g., a single group member in each host) and observed between-host variation in species composition (e.g., different winners occupying different hosts). Moreover, as in reef fishes, having different winners in different hosts could explain the observed diversity of microbial species at the host population level.

To this end, here we develop a computational framework to characterize the distribution of species across microbiome samples and to identify groups of microbial species whose distribution potentially reflects a competitive lottery schema. We defined the groups taxonomically as it has previously been found that the strongest priority effects occur between closely related bacteria [26]. Moreover, phylogenetically related species are more likely to have similar sets of genes and accordingly, similar niches. For example, it has been shown that a group of genes that includes many ABC transporters and two-component systems (which are involved in sensing nutrient levels and are likely related to niche space) is primarily conserved within the *Vibrionaceae* family but not in more distant relatives [33]. Similarly, a microscopy study has shown that different *Bacteroides* species overlap in spatial organization within the gut [34], further supporting the notion that closely related species are more likely to compete for a shared niche. Applying this framework to thousands of metagenomic samples from the gut microbiome, we found that indeed, different microbial clades follow different schemas and that while most of the common gut-dwelling microbial clades do not appear to follow the lottery schema, several less well-studied groups exhibit strong lottery-like-induced assembly patterns.

## Results

### Identifying competitive lottery-governed genera in the human gut microbiome

Our model of microbiome assembly assumes a collection of species, which are divided into several groups. These groups may represent phylogenetically related species or guilds of unrelated species that compete for similar niche space. Our model further assumes that the abundance of each species in the microbiome is determined by a two-step assembly process. The first step determines the abundance of each group based on, for example, the total niche space available to that group (Additional file 1: Figure S1A; note that when discussing abundances, we are always referring to “relative” abundances). Once the group abundance has been determined, a second step takes place where the abundance of each group is divided between the group’s species following a specific schema, which could be the competitive lottery schema (Additional file 1: Figure S1B) or some other unknown schema. These schemas reflect within-group ecological processes such as competition for this niche space or commensal interaction. Importantly, we assumed that different groups may be governed by different schema but that the same group is governed by the same schema in all samples (reflecting inherent ecological or functional attributes of that group). We further assumed that the two assembly steps are completely independent and focus on identifying the ecological processes that govern the second step of assembly.

To assess the applicability of the competitive lottery schema to the human gut microbiome, we obtained a dataset of 8638 gut microbiome samples that have been assayed using 16S rRNA sequencing from the American Gut project [35]. From this data we selected those samples with at least 5000 reads, resulting in a total of 7781 samples. The data had been clustered by QIIME [36] into operational taxonomic units (OTUs) using closed reference clustering. We further filtered OTUs with low abundance or that do not appear in many samples (see the “Methods” section). We additionally conducted a robustness analysis, demonstrating that various parameter choices in processing these data did not qualitatively impact our findings below (see Additional file 2: Supporting Text and Additional file 3: Figure S2A).

As noted above, we assumed that groups are defined phylogenetically and initially consider each genus as a single group (later expanding our definition to higher-level taxonomic groups). Indeed, phylogenetically related species are likely to have similar niches, tend to have similar gene content, and often have similar metabolic capacities, ultimately giving rise to intense within-group competition [18, 37, 38]. Furthermore, priority effects are strongest between phylogenetically related groups of species [26]. Clearly, such phylogenetic grouping may not capture all groups of microbes with a similar niche and in some cases unrelated species may form important functional guilds, yet such guilds are challenging to define rigorously and are therefore ignored in our analysis below.

To assess whether the distribution of OTU abundances within each genus reflects assembly by a competitive lottery schema, we note that under this schema, OTU distribution is expected to exhibit two key characteristics: (i) in each sample (or at least in most samples), the group’s abundance is expected to be captured primarily by a single group member (the “lottery winner”) and (ii) different samples are expected to have different lottery winners. In our analysis below, we define lottery winners as OTUs that capture > 90% of the group abundance (and see Additional file 2: Supporting Text and Additional file 3: Figure S2B for justification and sensitivity analysis). Given this definition, we examined the distribution of OTU abundances within each genus and assessed the two characteristics above by calculating two measures: (i) *winner prevalence*—the fraction of samples in which one OTU was assigned > 90% of the genus abundance—and (ii) *winner diversity*—the normalized diversity of lottery winners (see the “Methods” section for complete details).

**Fig. 1:**
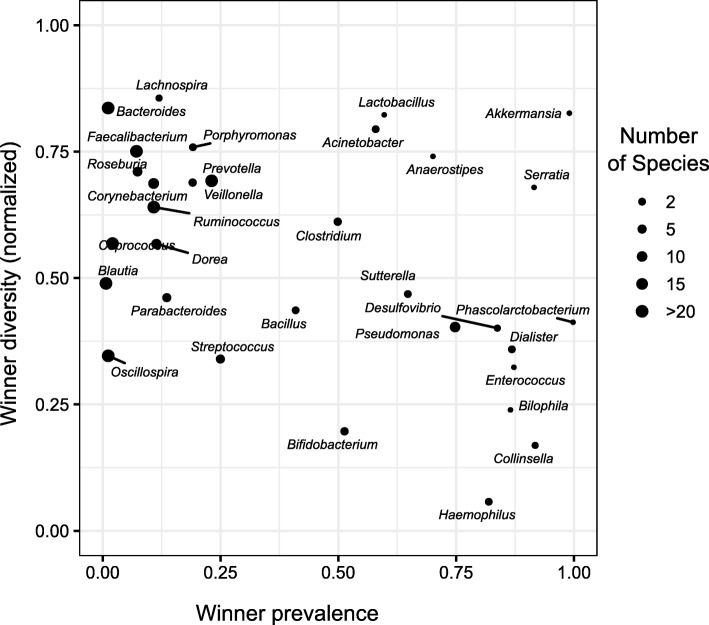
A scatter plot of the winner prevalence and winner diversity for different genera in the American Gut data. The winner prevalence is defined as the fraction of samples in which a winner OTU (an OTU occupying more than 90% of the group’s abundance) is observed. The winner diversity is defined as the Shannon diversity of the winner distribution (i.e., the frequency as which each OTU occurs as the lottery winner among all samples in which a lottery winner is observed). The winner diversity was normalized by the maximum diversity (log_2_ of the number of winners, representing the Shannon diversity if all winners are observed at the same frequency) and hence range from 0 to 1. A low diversity suggests that the same OTU occurs as the lottery winner in all samples, while a high diversity suggests a more even frequency of different OTUs as lottery winners

Plotting these two measures for each genus revealed several intriguing patterns (Fig. 1). Specifically, we found a number of genera with very high winner prevalence (i.e., where a lottery winner is observed in the vast majority of samples). For example, in *Akkermansia*, 99% of samples have one OTU occupying more than 90% of the group’s abundance (and in fact, in 94% of samples, the winner OTU occupies more than 99% of the group abundance). Similarly, in *Serratia*, in 91% of the samples, one OTU captures > 90% of the abundance. Interestingly, some of the genera with a high winner prevalence represent relatively poorly studied members of the gut microbiome, such as *Phascolarctobacterium* which is a non-typical gram-negative member of the gram-positive Firmicutes phylum. In contrast, most well-studied genera in the gut microbiome (e.g., *Bacteroides* and *Prevotella* from the Bacteroidetes phylum and *Faecalibacterium*, *Blautia*, and *Oscillospira* from the Firmicutes phylum) have a relatively low winner prevalence, with only a few (< 25%) of the samples having a single OTU capturing > 90% of the group abundance. While the assembly of these taxonomic groups could be governed by a number of different mechanisms, these findings suggest that it does not involve complete competition-derived exclusion. We further examined the relationship between the winner prevalence and the number of OTUs in the group. Naturally, the smaller the number of OTUs in a group, the more likely it is that one of these OTU reaches > 90% abundance, and indeed, there is some correlation between these two properties (Additional file 4: Figure S3). Yet, there are some groups with many OTUs that exhibit high winner prevalence (e.g., *Pseudomonas* that includes 19 OTUs and still have winner prevalence of 0.75) and others with relatively few OTUs that exhibit low winner prevalence (e.g., *Veillonella* that includes 6 OTUs and still have winner prevalence of 0.19). Moreover, repeating this analysis while considering only the 3 most abundant OTUs in each genus suggested that while the number of species plays a role in winner prevalence, it does not account for the observed separation between low and high winner prevalence genera (Additional file 2: Supporting Text, Additional file 3: Figure S2C, and see also Additional file 5: Figure S4 below). Notably, we found no evidence suggesting that our identification of competitive lottery groups has been affected by the group rarity or low abundance (Additional file 4: Figure S3).

Next we turned to examine the winner diversity of the various genera, focusing primarily on genera with high winner prevalence. As expected, some of these genera (e.g., *Haemophilus*) exhibit extremely high winner prevalence but extremely low winner diversity (Fig. 1). Put differently, each of these genera is dominated by the same high-abundance OTU across all samples and is accordingly inconsistent with the competitive lottery schema. We also observed that groups with low winner prevalence generally have comparatively higher winner diversity, likely reflecting sampling effects. Importantly, however, some high winner prevalence genera (e.g., *Akkermansia* and *Serratia*) also exhibit high winner diversity, reflecting different winners in different samples, as expected by the competitive lottery schema. Notably, the lottery schema does not necessarily entail maximum winner diversity (i.e., winner diversity score of ~ 1, reflecting a scenario where all winners occur at the same frequency), but rather a plurality of winners at potentially different frequencies (and see examples below).

**Fig. 2:**
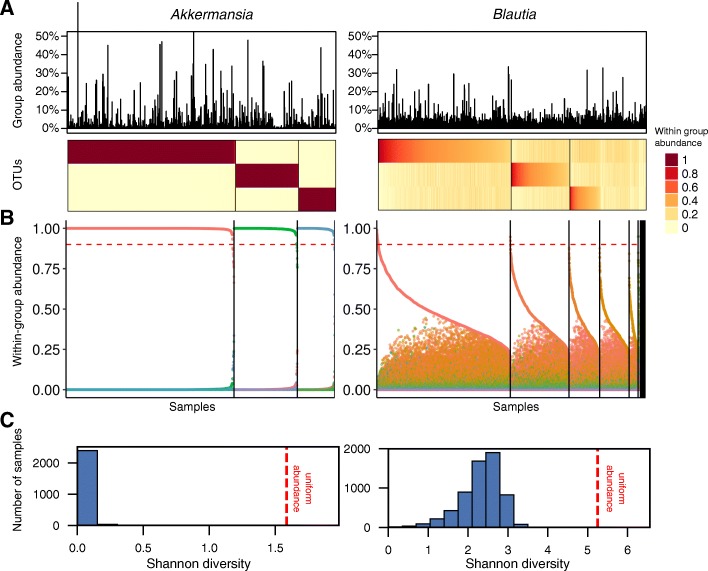
Distribution of within-group abundances in a representative lottery-like genus (*Akkermansia*) and in a non-lottery-like genus (*Blautia*). **a** Heatmaps showing the normalized within-group abundance of OTUs in the *Akkermansia* and *Blautia* genera. Here we only show the three most abundant OTUs of *Blautia* for ease of comparison with *Akkermansia.* The bar plots on the top indicate the sum of the genus abundance in each sample. Only samples in which the genus’ abundance is > 0.5% were included. Samples have been ordered first by the identity of the most abundance OTU (with vertical lines separating set of samples with different most abundant OTU) and second within each such set of samples by decreasing abundance of that most abundant OTU. **b** Point plots showing the normalized abundance of OTUs in each genus across samples. Samples have been ordered in an identical way to that described in **a**. The dashed red line denotes the 0.9 cutoff used to define lottery winners. In contrast to **a**, here we included all OTUs in the group. **c** A histogram displaying the Shannon entropy of the within-group abundances within each sample for *Akkermansia* and *Blautia*. The red dotted line corresponds to the Shannon diversity in an idealized group in which the abundances of all OTUs are uniformly spread

**Fig. 3:**
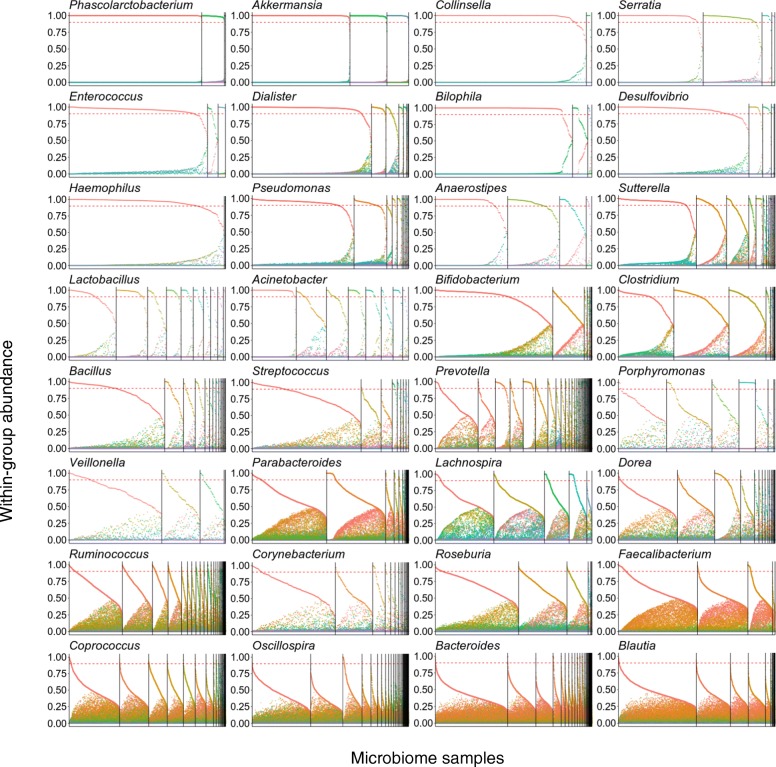
Distribution of within-group abundances for all genera. Details are as in Fig. 2b. Genera are sorted by decreasing winner prevalence

To more closely explore the structure of genera with high vs. low winner prevalence (and high vs. low winner diversity), we further examined the distribution of OTUs in each genus, highlighting the different patterns governing OTU abundances (Fig. 2). In *Akkermansia* (a genus identified above as having a high winner prevalence and high winner diversity), for example, a single OTU clearly occupies the majority of this genus’ abundance in each sample and the lottery winner OTU varies from sample to sample, as predicted by the competitive lottery schema (Fig. 2a). This is in sharp contrast to genera with very low winner prevalence such as *Blautia*, where the group abundance is more evenly distributed among the group OTUs (note that in Fig. 2a, samples and OTU are ordered to emphasize any potential lottery pattern). The difference in OTU distribution between these two genera is even clearer when visualizing the fraction of the group abundance captured by the most abundant OTU in the sample and comparing it to the abundance of the other OTUs (Fig. 2b). Indeed, in *Akkermansia*, the most abundant OTU in each sample generally captures ~ 100% of the group’s abundance, whereas in *Blautia*, the most abundant OTU generally coexists with other OTUs (Fig. 2b). We again confirmed that this is not an artifact of the higher number of OTUs included in *Blautia* compared to *Akkermansia* (see Additional file 2: Supporting Text and the plots for these two genera in Additional file 5: Figure S4). To further quantify this effect, we calculated the Shannon diversity observed in each sample within these genera, again demonstrating a markedly skewed distribution toward low diversity (namely a single OTU) in *Akkermansia* compared to a more even distribution observed in *Blautia* (Fig. 2c). Examining the distribution of OTU abundances across all genera further revealed both additional clear cases of lottery-based assembly and more complex patterns (Fig. 3 and Additional file 5: Figure S4). For example, OTU abundance distributions in *Phascolarctobacterium*, *Serratia*, and *Dialister* exhibit all the hallmarks of lottery-based assembly, including complete exclusion and high diversity of winners. In contrast, in *Acinetobacter* and in *Porphyromonas*, some OTUs show nearly complete exclusion, whereas others show very little exclusion. Finally, the OTU distribution in *Haemophilus* clearly reflects the single lottery winner suggested by the genus’ low winner diversity reported above.

### Identifying higher-level competitive lottery-governed taxonomic groups

To more comprehensively characterize the ecological processes that are at play in the assembly of the gut microbiome, we next examined whether a similar lottery schema may govern the assembly of higher-level taxonomic groups. Indeed, our initial focus on genus-level groups was arbitrary, and a broader characterization of taxonomic groups across the microbial tree of life can provide a more complete picture of the assembly mechanisms of the human gut microbiome. Such a characterization will also allow us to examine the consistency of assembly schemas across different taxonomic lineages and to identify interesting patterns in the evolution of assembly rules.

To this end, we extended our model above to assess the assembly schema of each taxonomic group in a hierarchical manner. Specifically, just as we had assessed the winner prevalence and winner diversity for genus-level groups of OTUs, we assessed the winner prevalence and winner diversity for higher order taxonomic groups (e.g., a specific family) by looking at the abundance of different *subgroups* of that group (e.g., a single genus) and the abundance of each subgroup in each sample. Notably, with this definition, we considered the abundance of the subgroup regardless of how its abundance is distributed among the subgroups’ members (and accordingly regardless of the winner prevalence and winner diversity of the subgroup itself). Put differently, when calculating these parameters for a given group, we considered each of its subgroups as a single entity (whose abundance is simply the sum of abundances of the subgroup’s member).

**Fig. 4:**
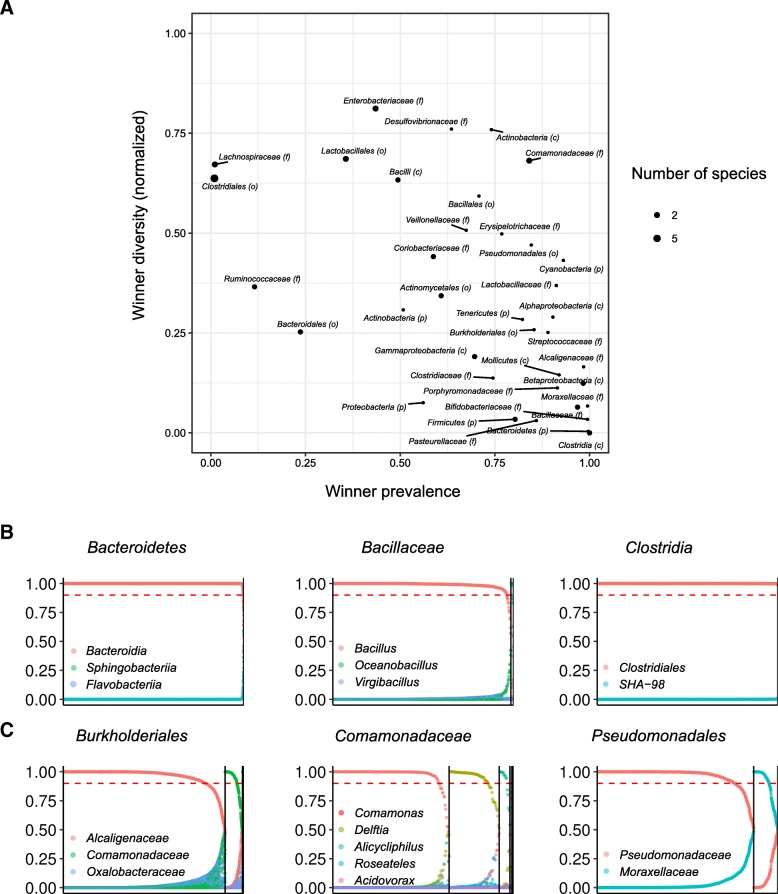
Winner prevalence, winner diversity, and subgroup abundance distribution for higher-level taxonomic groups. **a** A scatter plot of the winner prevalence and winner diversity for different taxonomic groups in the American Gut data. Details are as in Fig. 1, but considering the abundances of subgroups (rather than OTUs) within each taxonomic group. The taxonomic order of each groups is in parentheses next to the group (p phylum, c class, o order, f family). **b** Examples of higher-level taxonomic groups that can only maintain a single winner (and thus, low winner diversity). **c** Examples of higher-level taxonomic groups that have a diversity of lottery winners. Details are the same as in Fig. 2b

Applying this extended method to the dataset described above and examining taxonomic groups at varying levels up to the phylum level revealed complex and intriguing patterns of community assembly (Fig. 4). Notably, at higher phylogenetic levels, we found many groups with high winner prevalence but low winner diversity (Fig. 4a and compare with Fig. 1), reflecting a single dominant subgroup. For example, the phylum Bacteroidetes, the class *Clostridia*, and the family *Bacillaceae* are each dominated by a single subgroup (the class *Bacteroidia*, the order *Clostridiales*, and the genus *Bacillus*, respectively) in nearly all samples (Fig. 4b). Yet, several groups at these higher phylogenetic levels, including the orders *Burkholderiales* and *Pseudomonadales* and the family *Comamonadaceae*, again exhibited the hallmarks of a lottery-based assembly, with both high winner prevalence and high winner diversity (Fig. 4a). Indeed, examining the distribution of subgroups in these lottery-like groups clearly demonstrates that only a single subgroup from each group dominates each sample, but that different samples are dominated by different subgroups (Fig. 4c). Other groups exhibited strong coexistence with multiple subgroups co-occurring in each sample (e.g., the order *Clostridiales*) or more complex assembly combining both exclusion and coexistence patterns (e.g., the phylum *Proteobacteria*; Additional file 6: Figure S5).

Note also that our definition above allows for complex hierarchical patterns of assembly schemas (Additional file 7: Figure S6). For example, the order *Lactobacillales* exhibits clear coexistence patterns with multiple families from this order co-occurring in each sample, yet some of these families (e.g., *Lactobacillaceae* and *Streptococcaceae*) in turn exhibit lottery-like assembly, with only one genus from each family present in each sample (Additional file 6: Figure S5 and Additional file 7: Figure S6). Furthermore, while the family *Streptococcaceae* exhibits lottery-based assembly, with the genus *Streptococcus* generally excluding the genus *Lactococcus*, the different OTUs in the genus *Streptococcus* tend to coexist (see Fig. 3).

### Assembly schemas are consistent across multiple datasets

**Fig. 5:**
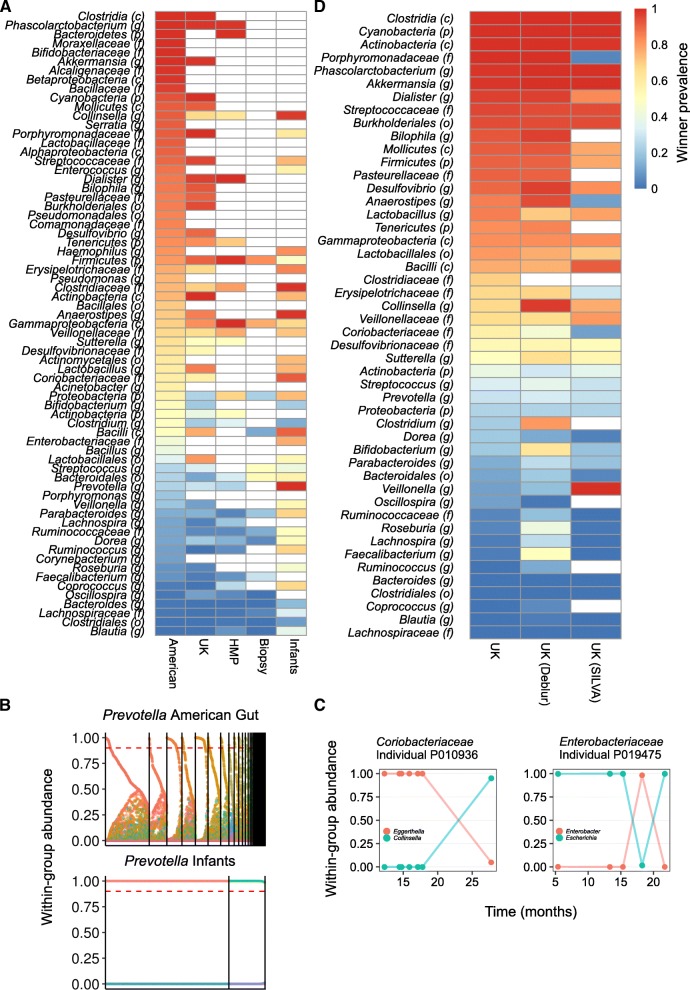
Robustness of winner prevalence parameter. **a** A heatmap showing the winner prevalence parameter for each group across multiple independent datasets. Groups that are missing in a given dataset are displayed as white cells. The taxonomic order of each group is in parentheses next to the group name as in Fig. 4a. **b** The within-group abundance of members of the genus *Prevotella*. Details are as in Fig. 2b. **c** The within-group abundance of members of *Coriobacteriaceae* and *Enterobacteriaceae* in two infant individuals with lottery winner turnover. Both individuals are found in [42]. **d** A heatmap showing the winner prevalence in the UK twins dataset when processed by QIIME with GreenGenes annotations (left), when processed by Deblur with GreenGenes annotations (middle), and when processed by QIIME with SILVA annotations (right). Details are the same as in Fig. 5a

We next set out to confirm that our findings are not specific to the American Gut data due to cohort-specific population structure or study-specific protocols. For example, in the American Gut project, samples have been collected without freezing, in contrast to most other large-scale microbiome studies. Such differences in sample preservation could impact growth conditions post-egestion and ultimately affect observed within-group abundance distributions. Similarly, different sequencing methods or different computational processing pipelines could impact inferred community compositions and introduce bias into the estimated lottery parameters. To this end, we further obtained several other microbiome datasets, characterized the assembly of phylogenetic groups in each such dataset, and compared the obtained lottery parameters across datasets (Fig. 5). Notably, different datasets may include a somewhat different set of OTUs and accordingly a different set of groups that can be compared given our filtration process. We first considered an independent 16S-based gut microbiome dataset, obtained from a twin study in the UK [39]. We focus on this dataset because of the large number of samples (*n* = 1017 using the same 5000 read cutoff as above). Calculating the lottery measures defined above for all groups in this dataset, we found a strong correlation between the lottery prevalence parameter in the American Gut data and in the UK twins data (Fig. 5a, *ρ* = 0.93; Spearman correlation test), suggesting that a similar suite of groups may be assembled according to a lottery-like model. We next considered the human microbiome project (HMP; [1]), which is commonly used as a benchmark for human microbiome studies. Again, we found a strong correlation between the lottery prevalence values calculated for the HMP and the American Gut dataset (Fig. 5a, *ρ* = 0.91). Since species interactions that give rise to a lottery-like distribution probably primarily occur between species that are at close proximity, we further considered a gut microbiome dataset that was obtained from biopsies [40] (rather than fecal samples), allowing us to evaluate whether our findings hold in microbiome profiles sampled from regional microenvironments in the gut. Even though this dataset is naturally substantially smaller (*n* = 23 after our filtration process), we still found a good correspondence between the winner prevalence parameter in these biopsy samples to those calculated based on the American Gut data (Fig. 5a, *ρ* = 0.85).

Finally, we considered microbiome data from several infant cohorts ([41–44] and see Methods). We again found a significant correlation between the winner prevalence parameter in this infant dataset and the adult data analyzed above (Fig. 5a, *ρ* = 0.53 for the American Gut data). Interestingly, however, we also found a number of notable differences between this infant dataset and the adult datasets. For example, *Prevotella* has a low winner prevalence in the American Gut, UK twins, and HMP datasets, but a much higher winner prevalence in the infant dataset (Fig. 5b), suggesting that while the developing gut microbiome in infants can support only a single species from that group, the adult microbiome can potentially support multiple coexisting species from that group. It is also worth noting that winner prevalence values are generally somewhat higher in the infant dataset compared to the adult datasets for many groups, potentially suggesting different assembly dynamics compared to adults. This is in agreement with various studies demonstrating that infant microbiomes are less stable and less diverse than the adult established microbiome [42, 45, 46]. To further explore these dynamics, and thanks to the multiple samples per individual available in the infant dataset, we set out to examine whether lottery winners are stable or transient in infants (“Methods” section). Examining the lottery winner over time, we found examples of lottery-winner turnover. For example, in one individual, the initial winner in the family *Coriobacteriaceae*—the genus *Eggerthella*—is later replaced by the genus *Collinsella*, while in another individual, a turnover in the family *Enterobacteriaceae* is later reverting back to the initial winner (Fig. 5c). However, such examples are relatively rare, with turnovers observed in only 35 out of the 287 examined individual-group pairs (“Methods” section). Notably, such turnovers were not observed in adult samples with multiple time points from the HMP dataset (out of 70 analyzed).

We also examined how the winner diversity parameter changes between these different datasets. Notably, this parameter is not necessarily expected to be highly correlated between different cohorts since the distribution of winners may depend on the prevalence of different taxa in these populations and may be more influenced by differences in sample collection and preservation. Indeed, while we found a significant correlation between the winner diversity values obtained for the American Gut and the UK datasets (*ρ* = 0.67, *P* < 5 × 10^−07^), comparison with other smaller datasets was not statistically significant. Nonetheless, many of the groups that exhibited high winner diversity (> 0.25) in the American Gut data also exhibited high winner diversity in other datasets (30 out of 38 in the UK dataset, 24 out of 28 in the infant dataset, and 18 out of 22 in the HMP dataset).

Finally, we sought to investigate whether our results are sensitive to the OTU processing approach and taxonomic annotations used (Fig. 5d). We focused on the UK twin dataset discussed above (which was originally clustered into OTUs by Qiita and annotated by the GreenGenes taxonomy) and examined whether using instead a sequence denoising approach (Deblur; [47]) or a different taxonomy (SILVA; [48]) impacts the obtained lottery parameters. We found a strong and significant correlation between the lottery parameters obtained in the original UK twin datasets to those obtained with sequence denoising or SILVA (lottery prevalence *ρ* = 0.93 and *ρ* = 0.77, respectively; lottery diversity *ρ* = 0.70 and *ρ* = 0.50, respectively), suggesting that our findings are not an artifact of a specific processing pipeline. Moreover, the few instances where groups displayed substantial differences in winner prevalence (e.g., *Porphyromonadaceae*, which exhibited high winner prevalence when annotated with GreenGenes but low winner prevalence when annotated with SILVA) appear to occur due to additional annotations to these groups in SILVA.

### Genomic determinants of group assembly

Why would the niche space available to lottery-like groups such as *Akkermansia*, *Dialister*, and *Phascolarctobacterium* only support a single OTU, while the niche space available to non-lottery-like groups such as *Bacteroides*, *Parabacteroides*, and *Faecalibacterium* allows multiple species to colonize the same community? One possibility is that coexisting species can exploit a more diverse set of resources and therefore partition their niche, allowing multiple species to inhabit it [18, 49]. Species in lottery-like groups, in contrast, may have a narrower and overlapping nutritional niche, promoting fierce competition and mutual exclusion. This hypothesis is also in line with previous theories concerning the different strategies specialists and generalists species may employ to compete for nutrients. For example, a recent large-scale metabolic modeling-based study of bacterial ecological strategies has demonstrated that metabolic variability is correlated with growth rate and with competition [50]. It suggested that microorganisms may adopt one of two strategies: a specialist strategy that is associated with little co-habitation (analogous to our lottery-like schema) or a generalist strategy that is associated with fast growth and intense co-habitation (analogous to our non-lottery-like schema). Similarly, an assay discussing oligotrophs vs. copiotrophs (organisms that thrive in nutritionally poor vs. rich environments, respectively) suggested that one of the key reasons underlying the different environments in which such organisms survive has to do with the efficiency with which they compete for certain nutrients [51].

Since the nutritional niche of most lottery-like group species has not been comprehensively characterized to date, we tested this hypothesis using genomic data, assuming that the size of a species’ genome can serve as a proxy for its ability to exploit a broad range of resources [18, 50, 52]. To facilitate this analysis, we linked each OTU to a reference genome from NCBI using BLAST (“Methods” section). We additionally obtained genome annotation data for these genomes from the Integrated Microbial Genomics database (IMG, [53]; Methods). To avoid the complexities associated with hierarchical taxonomic levels, we only considered genus-level groups. We then focused on genera that had high winner diversity (> 0.25, i.e., ignoring groups with a single fixed winner) and compared the genomes of species from genera with high (> 0.75) vs. low (< 0.75) winner prevalence in the American Gut dataset and at least one other of the datasets analyzed above.

**Fig. 6:**
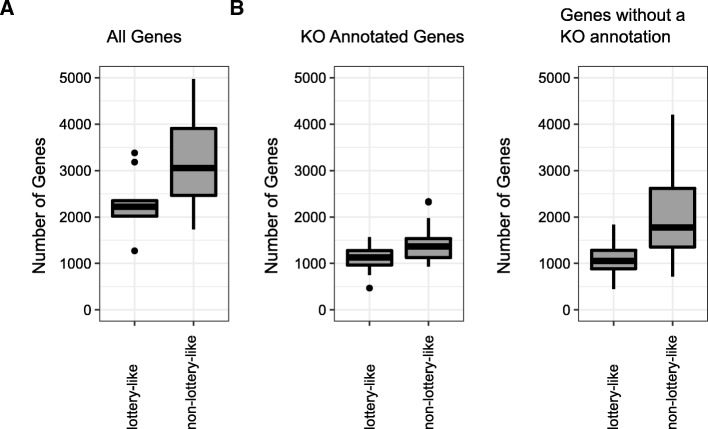
The number of genes in the genomes of species in groups with high winner prevalence. **a** Boxplot showing the distribution of the number of genes in the genomes of species from genera with high winner diversity (> 0.25) and with high winner prevalence (> 0.75) vs. low winner prevalence (< 0.75). Only genera that had at least one 16S sequence that could be assigned to a sequenced reference genome are included. **b** Equivalent plots to **a**, but with the number of genes in the genome split into genes with and without a KO annotation

This analysis demonstrated that species in lottery-like genera have significantly fewer genes compared to species in non-lottery-like genera (Fig. 6a; *P* < 0.005). This simple, yet important difference between species in lottery vs. non-lottery-like groups is in agreement with our hypothesis above, suggesting that competitive lottery groups tend to represent more specialized and streamlined species that cannot partition their niche. Furthermore, we found that this difference in the number of genes is not uniform for all genes and that it is much more pronounced for genes *without* a KO annotation, suggesting that the coexistence of species in non-lottery-like groups may involve novel and yet-to-be-characterized mechanisms (Fig. 6b; *P* < 0.05 for KO-annotated genes, *P* < 0.005 for genes with no KO annotation).

## Discussion

In this paper, we used a simple model for the assembly of the human gut microbiome to assess how well different groups follow the competitive lottery schema. We found that the canonical microbiome members from the Bacteroidetes and Firmicutes phyla do not appear to be governed by a lottery-like schema but rather support a non-lottery-like assembly that supports significant coexistence among members. This result is perhaps not surprising given the current state of knowledge of the microbiome and numerous studies that report various species coexistence patterns and analyses [54]. Importantly, however, we did find a substantial number of taxonomic groups that exhibit lottery-like distribution, including the genera *Akkermansia*, *Dialister*, and *Phascolarctobacterium*. In these lottery-like genera, the entirety of the group’s abundance quota is occupied by a single OTU (though this OTU may be different in each sample), and all other members are excluded from the community. Notably, a few of the identified lottery-like groups, such as *Phascolarctobacterium*, tend to be understudied compared to the non-lottery-like groups and relatively little is known about their ecology, niche, and interactions with other species. Other lottery-like groups are relatively well characterized, such as *Akkermansia*, whose members are metabolically flexible and play a role in the host metabolic health [55]. Moreover, some of the identified competitive lottery groups have unusual properties. For example *Dialister* and *Phascolarctobacterium* from the *Veillonellaceae* family are gram-negative members of the gram-positive Firmicutes phylum, and yet form endospores, which is generally considered a gram-positive trait [56].

Why might some taxonomic groups allow for multiple members to occupy a given host, while in others a single species outcompetes and excludes other members of the group? Ecological theory posits that species can coexist within a host (or a “patch”) when stabilizing niche differences are greater than relative fitness differences [49]. Restricting our attention to stabilizing niche differences and particularly resource partitioning, we can consider the niche of a species as the set of nutrient metabolites that it can grow on, as postulated by the nutrient niche model [57]. Given this model, it is easy to see how a non-lottery-like group such as the *Bacteroides* has partitioned resources between related species. Specifically, *Bacteroides* are known sugar metabolizers and have experienced an incredible expansion in the number of polysaccharide utilization loci (PULs) encoded in their genomes [58]. In *Bacteroides thetaiotaomicron* for example, these genes constitute 18% of the genome [59]. This expansion likely allows the exploitation of a diverse array of nutrients since different glycoside hydrolase (GH) enzymes encoded in these PULs are specific for different bonds in glycan polymers [60]. The presence of different PULs in different species could accordingly promote diversification in the set of nutrients each species utilizes and ultimately permit coexistence. Indeed, in vitro work suggests that different species of *Bacteroides* prefer different glycans as a food source and preferentially transcribe corresponding PULs, enabling coexistence [61]. With this hypothesis in mind, lottery-like groups that appear to be able to maintain only a single representative per sample likely prohibit coexistence since they are unable to engage in resource partitioning. For example, it has been suggested that carbohydrate degradation might be a part of the niche of *Megasphaera* [62], a genus that we have identified as being likely governed by the lottery schema. Moreover, examining the genomes of *Megasphaera* species, we note that they encode on average only 4.25 GH enzymes, compared to 192 GH enzymes on average encoded by members of the non-lottery-like group *Bacteroides* (data not shown), further supporting the narrower niche of competitive lottery groups.

These observations are in agreement with our findings of different genome size in lottery-like vs. non-lottery-like groups. Expanded gene content in the non-lottery-like groups could allow for niche diversification around a common theme of nutrients, such as through PUL expansion in *Bacteroides*. Similarly, the non-lottery-like groups from the Firmicutes phylum appear to be generalists based on their genomic content [38] and thus have ample opportunity for niche diversification. Lottery-like groups such as *Akkermansia*, *Dialister*, and *Phascolarctobacterium* on the other hand are likely existing on a very narrow set of nutrients without any such opportunities for resource partitioning, hence their smaller genomes. This is consistent with previous findings that generalists have larger genomes than specialists [63]. Extreme cases of narrow niches are associated with exceptionally small genomes such as the less than 1000 genes in the epibiotic TM7x [64]. This hypothesis is also in agreement with the trend toward a larger number of genes without a KO annotation in the non-lottery-like groups, which likely are playing a disproportionately larger role in niche diversification compared to genes with KO annotations that are more commonly involved with fundamental cell biology and core metabolism. It should also be noted that our study has focused on phylogenetically defined groups, and accordingly, our identified lottery-like groups represent potential competition between closely related species. Indeed, a model-based study of predicted metabolic environments has found a substantial agreement between phylogenetic relatedness and similarity in nutritional requirements [65]. Yet, some taxonomic groups may experience *stronger* competition from phylogenetically distant but metabolically similar taxa (e.g., due to convergent evolution), potentially outweighing within-group competition and accordingly exhibiting non-lottery-like patterns.

Interestingly, in the original formulation of the competitive lottery model in reef fishes, patches are dominated by a single fish and this enables coexistence of multiple fish species across the entire reef. By analogy, in the microbiome ecosystem, each host corresponds to a patch and the population of hosts corresponds to the entire reef. With this in mind, competitive lottery-based exclusion within each host might lead to maintenance of competing and functionally similar species at the host population level and ultimately to a larger pool of diverse species across the population.

Notably, with the strictest view of the competitive lottery schema, it is unlikely that an established strain can be eliminated from the community, since strong priority effects exclude all invaders from the same group. Yet, given the instability associated with the developing microbiome of an infant gut, our finding of lottery winner turnover in this setting and the imperfect fit to the ideal competitive lottery schema are perhaps not completely unexpected. Previous work has found that the gut community approaches an adult-like composition by 12 months of age, but is generally considered to continue developing until 36 months of age [42, 43]. Indeed, a previous study of *Bacteroides fragilis* found that the dominant *B. fragilis* strains are not stable in the developing infant gut and can be replaced by other strains throughout the course of development [46], and similar dynamics have been observed in other species [42].

Recent years have witnessed multiple studies aiming to characterize co-occurrence relationships in the human gut microbiome [8, 9, 66]. Our group-based assembly model provides a complementary approach to such co-occurrence studies for understanding community composition. While there are some similarities between our analysis approach and co-occurrence detection methods, they differ in several elements and in the underlying assumptions made. Most importantly, co-occurrence methods focus on interactions between *pairs* of OTUs whereas our approach assesses interactions within an entire *group* of OTUs. As such, the biological insight gained from co-occurrence studies tends to relate to pairwise biochemical dependencies [9] or cross-feeding interactions [18, 67, 68], while the insight from our study relates to the ecological processes acting on a group of organisms as a whole.

It is also worth noting that our model implicitly makes a number of assumptions about the forces and processes at work during microbiome assembly. First, since we are using a group-level assembly model, most assumptions relate to the importance of groups in the microbiome. Specifically, we assume that species are part of guild-like groups and that these groups are the primary determinant of how community assembly occurs. Furthermore, we assume that microbial interactions occur primarily within the groups and that between-group interactions do not impact without group interactions and assembly. We also assume that groups are governed by a coherent ecological assembly model that applies to every member of the group in an identical way. With regard to our reliance on metagenomic data (mostly 16S), we additionally assume that such data provide reasonable estimation of each species’ abundance, ignoring potential noise in abundance estimation. This is clearly not ideal, but since the competitive lottery schema entails an order of magnitude difference between the abundance of the lottery winner and the abundances of other species in the group, such metagenomic-based abundance estimates are likely sufficient for most groups. Moreover, since noisy abundance estimation likely has the strongest impact on very low abundance groups (where sampling error could be substantial), we used a simulation study to determine a reasonable OTU inclusion cutoff (Additional file 8: Figure S7) and only considered groups with high enough abundance.

Our analysis has focused on testing a simple assembly schema and on identifying groups that are likely governed by the lottery schema. Importantly, however, there are likely many other processes that are at play in the assembly of the human gut microbiome. Moreover, while the competitive lottery assembly schema assumes that strong priority effects lead to complete exclusion of all species beyond the first, it is important to note that priority effects can also have different outcomes. For example, positive priority effects, where a colonizing species aids subsequent species to join the ecosystem, may give rise to highly structured communities. For example, the plant *Jacobaea vulgaris* alters the soil conditions allowing other plant species to flourish [69]. There could also be a complex mixture of positive and negative priority effects that depend on the exact species of the group. For example, the oral bacterium *Porphyromonas gingivalis* usually cannot grow in the presence of *Streptococcus oralis*, but it is able to coexist with *S. oralis* in the presence of *S. gordonii* [70]. Furthermore, our hierarchical method shows that assembly schema can impose different structuring forces at different taxonomic levels, and thus, identifying the scope at which any given assembly schema can function is an avenue for future research.

## Conclusions

Beyond the findings described above, our study demonstrates the utility of a computational approach for understanding assembly processes in the human gut. Specifically, by assessing the competitive lottery schema and its fit to observed distribution of species abundances across samples, we were able to identify taxonomic groups that appeared to conform to this schema and others that diverged significantly from it. Future studies following this approach could further assess additional processes that are governing the assembly of the microbiome and their contribution to microbiome composition.

## Methods

### Species abundance data

We downloaded processed 16S rRNA data from the Qiita database which has applied QIIME to detect closed reference OTUs at the 97% identity level [36]. Obtaining data from Qiita ensured uniformity of preprocessing across samples and datasets and in a way that is in harmony with the practices of the field. Specifically, we downloaded data from the American Gut project [35], a large study of twin microbiomes from the UK [39]. Finally, we obtained a dataset of biopsy samples from the human colon [40], as well as data from the Human Microbiome Project [1], and applied QIIME to detect closed reference OTUs at the 97% identity level. We removed samples with less than 5000 16S counts and filtered any OTU that did not appear at > 0.05% abundance in 0.5% of samples (to a minimum of 10 samples), resulting in a total of 1514 OTUs across 7781 samples from the American Gut project, 1201 OTUs across 1017 samples from the UK twins study, 793 OTUs across 284 samples from the HMP dataset, and 78 OTUs across 23 samples for the biopsy data for downstream analysis. To focus on taxonomic groups for which statistical analysis could be robust, we further filtered groups that did not have > 0.5% abundance in at least 0.5% of samples (to a minimum of 10 samples). Furthermore, due to the large number of groups in the American Gut data, for simplicity, only groups that had > 0.5% abundance in > 200 samples were included in our analysis.

We defined groups of OTUs using the lineage assignments that had been precomputed in Qiita from QIIME’s closed reference mapping to GreenGenes [71]. OTUs were assigned to groups based on the genus that was assigned, and OTUs that could not be assigned to a known genus were removed from genus-level analysis. When working with MetaPhlAn data, we used the taxonomy that had been assigned to each species.

To evaluate the robustness of our findings to OTU preprocessing and annotation, we obtained a copy of the UK twins data that had been processed using a sequence denoising approach by Deblur [47] via Qiita. We additionally obtained the original sequence reads from this database, clustered them into OTUs using QIIME, and taxonomically annotated the obtained OTUs using the SILVA database [48]. Cutoffs were applied as described in the previous paragraph.

We also obtained shotgun metagenomic samples from infant microbiomes that were sampled over time. These were amalgamated from a number of different studies including a study of vertical inheritance [41], a study of autoimmune diseases [42], a study of antibiotic usage [43], and a study of the development of type 1 diabetes [44]. MetaPhlAn 2.0 was run on these samples to assess species-level abundance with default parameters. Using similar filtering criterion as with the 16S data described above resulted in 437 OTUs across 171 samples for downstream analysis. In our turnover analysis, we only considered those individuals with at least two time points with at least 0.5% group abundance for each group, resulting in 287 individual-group pairs in the infant dataset and 70 in the HMP dataset for downstream analysis. For these individual-group pairs, we looked for at least two time points with different lottery winners dominating the community (> 90% abundance). However, when visualizing the data, we kept the full complement of time points from that subset of individuals in our analysis.

### Assessing characteristics of lottery-like species distribution

Our model aims to describe the observed abundance of the most prevalent OTUs across microbiome samples based on an assembly process such as the competitive lottery schema. We assume that this assembly process determines the abundances of OTUs within a given group and that the same group follows the same process in all samples. Specifically, we assume a two-step model: in the first step, the total abundance of a sample (100%) is allocated between groups according to some unknown process. Then in the second step, the abundance allocated to each group is split between the group’s members according to a competitive lottery schema.

Given a pre-defined group of species, we quantified two parameters that relate to the competitive lottery assembly schema. The first parameter is how often species distribution within a group includes a lottery winner, which we define as a group member that captures > 90% of the group’s abundance. This cutoff was chosen based on a null model for species abundances assuming a stick breaking process (see Additional file 2: Supporting Text). The second parameter is a measure of the diversity of lottery winners and is calculated by the Shannon diversity of the distribution of winners across samples (i.e., the frequency as which each OTU or subgroup occurs as the lottery winner among all samples in which a lottery winner is observed). The winner diversity was normalized by the maximum diversity obtained when all winners are observed at the same frequency (= log_2_ of the number of winners) and hence range from 0 to 1. A low diversity suggests that the same OTU or subgroup occurs as the lottery winner in all samples, while a high diversity suggests a more even frequency of different OTUs or subgroups as lottery winners.

To determine the assembly schema of groups at higher taxonomic level than genera, we applied a similar approach considering the aggregated abundance of each subgroup as if it were a single OTU. For example, when evaluating the schema governing each family, we considered the aggregated abundance of each of its genera. OTUs without annotation to a given subgroup have been discarded to avoid combining the abundance of OTUs from two different unknown lineages. If there was only a single subgroup in a given group, we refrained from the analysis of a single member group and progressed to the next level of the tree.

In analyses that required classifying groups into lottery-like vs. non-lottery-like groups, we defined lottery-like groups as those that exhibit winner prevalence > 0.75 and winner diversity > 0.25. In our analysis of genome content, we specifically focused on groups that exhibit lottery-like assembly in both the American Gut dataset and at least one other dataset of those analyzed above.

### Genome analysis

We obtained 16S sequences from the partial or complete genomes of 76,657 bacteria from NCBI. In order to map OTUs from a group to sequenced genomes, we used BLAST against the database of 16S sequences and accepted the best hit above 97% identity. We then obtained information on gene content on those genomes from Integrated Microbial Genomics (IMG) [53] and averaged gene content number over each species.

## Additional files

Additional file 1: **Figure S1.** A conceptual illustration of the competitive lottery assembly model. In the first stage, the total abundance of each sample (100%) is split between a set of pre-defined groups. In the second stage, each group’s abundance allocation is split between its subgroups according to the competitive lottery schema where a single subgroup receives the majority of the group’s abundance allocation. (PDF 51 kb)

Additional file 2: Supporting text. (PDF 185 kb)

Additional file 3: **Figure S2.** Robustness of the winner prevalence estimate to different cutoffs. Bar plots show how the winner prevalence value changes for different genera if the cutoff was changed for OTU inclusion (A), for the lottery winner determination (B), and when only the three most abundant OTUs in each genera are considered (C). (PDF 33 kb)

Additional file 4: **Figure S3.** Number of OTUs and abundance of the genera that were analyze in Fig. 1. The left panel illustrates the winner prevalence of each genus (as reported in Fig. 1). The middle panel illustrates the number of OTUs in each group (after filtration of rare OTUs; see the “Methods” section), as well as the number of OTUs that have been lottery winners (> 90% of the group abundance) in at least one sample. Finally, the right panel illustrates the distribution of the overall group abundance across all samples in the American Gut data. (PDF 274 kb)

Additional file 5: **Figure S4.** Distribution of within-group abundances for all genera when considering only the three most abundant OTUs in each genus. Details are as in Fig. 3. (PDF 772 kb)

Additional file 6: **Figure S5.** Distribution of within-group abundances for higher-level taxonomic groups. Details are as in Fig. 4b, c. (PDF 813 kb)

Additional file 7: **Figure S6.** A taxonomic tree, with assembly parameters displayed as pie charts. On each group that was analyzed in our study, we display the proportion of samples where different group members were lottery winners (> 90% abundance) using different colors. The proportion of samples without a winner is illustrated in white. With this visualization, the winner prevalence parameter is therefore denoted by the proportion of non-white pie chart, and the winner diversity parameter is proportional to the number and distribution of different colors in the pie chart. Groups without a pie chart were only used as subgroups to groups with pie charts. The tree was created with the interactive tree of life [72]. (PDF 73 kb)

Additional file 8: **Figure S7.** Winner prevalence estimates in simulated groups at uniform abundance. Abundances have been simulated using a Poisson distribution assuming that OTUs are at a variable minimum abundance threshold (*x*-axis). If abundance estimates were perfect, we would expect a winner prevalence of zero, but noise associated with the sampling processes creates artificial winners. The dashed red line is the minimum abundance threshold used in our study. (PDF 18 kb)

## Abbreviations

HMP: Human Microbiome Project
IMG: Integrated Microbial Genomics
OTU: Operational taxonomic unit
PUL: Polysaccharide Utilization Loci
