# Supplementary figures and images for: Competitive lottery-based assembly of selected clades in the human gut microbiome
Source: Microbiome. 2018 Oct 19;6:186. doi: 10.1186/s40168-018-0571-8 (PMC6195700; doi:10.1186/s40168-018-0571-8)

Stage 1: Total abundance is split between groups

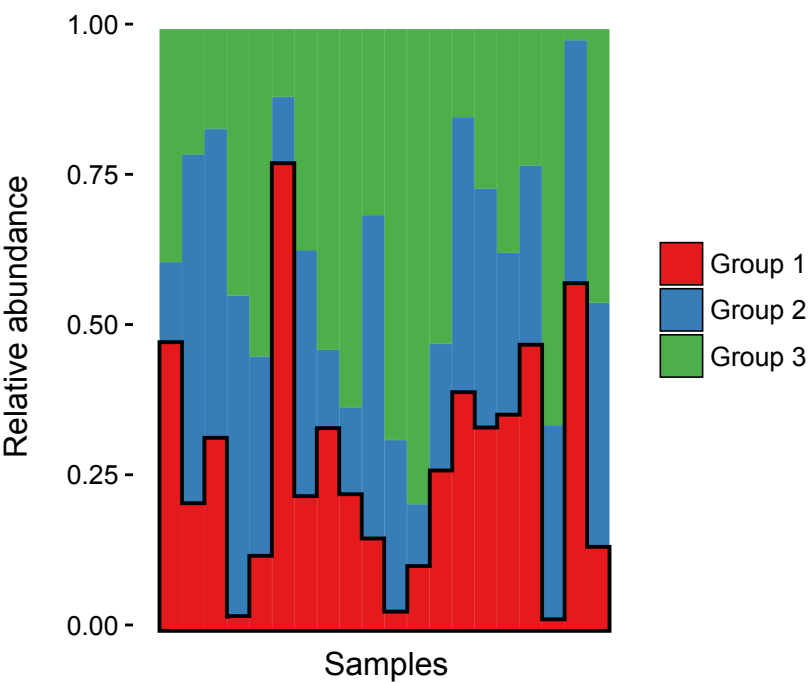

Stage 2: Group abundance is split between OTUs

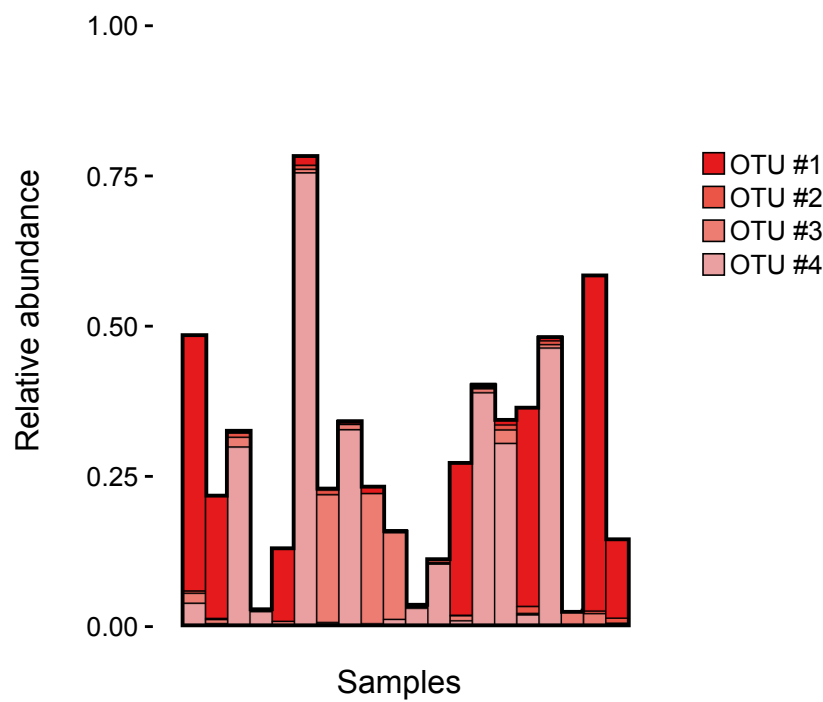

Supplement: Supplementary file 1 — Figure S1. A conceptual illustration of the competitive lottery assembly model. In the first stage, the total abundance of each sample (100%) is split between a set of pre-defined groups. In the second stage, each group’s abundance allocation is split between its subgroups according to the competitive lottery schema where a single subgroup receives the majority of the group’s abundance allocation. (PDF 51 kb) [file 40168_2018_571_MOESM1_ESM.pdf]

A

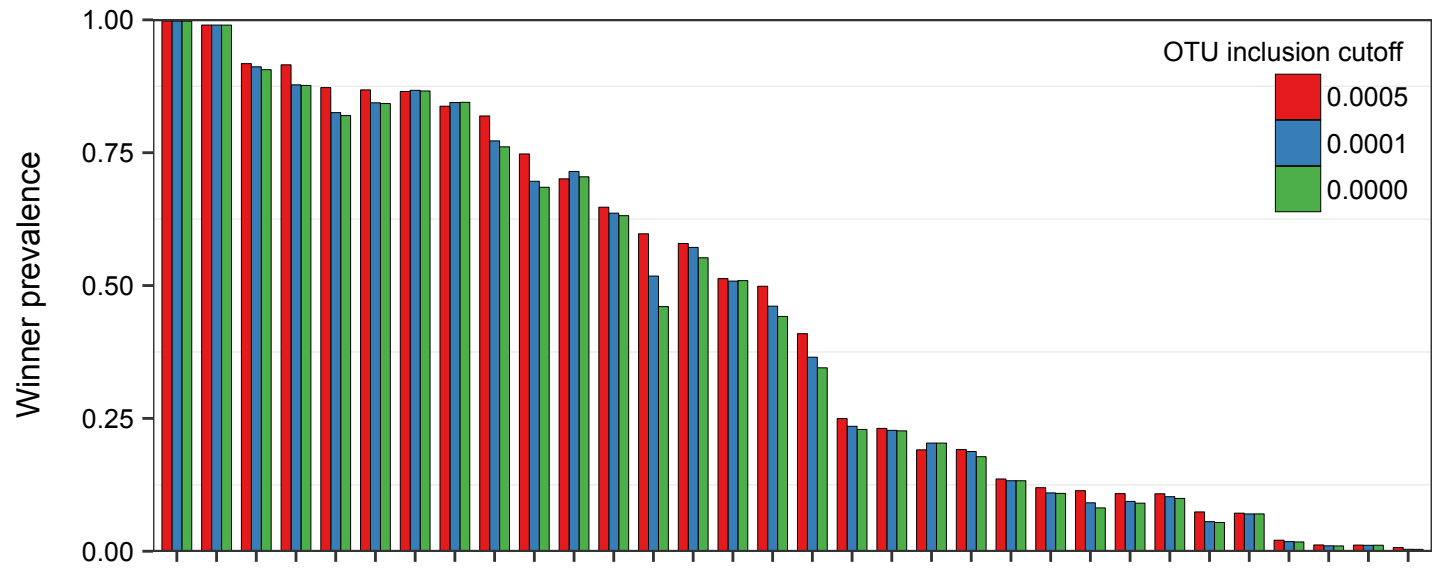

B

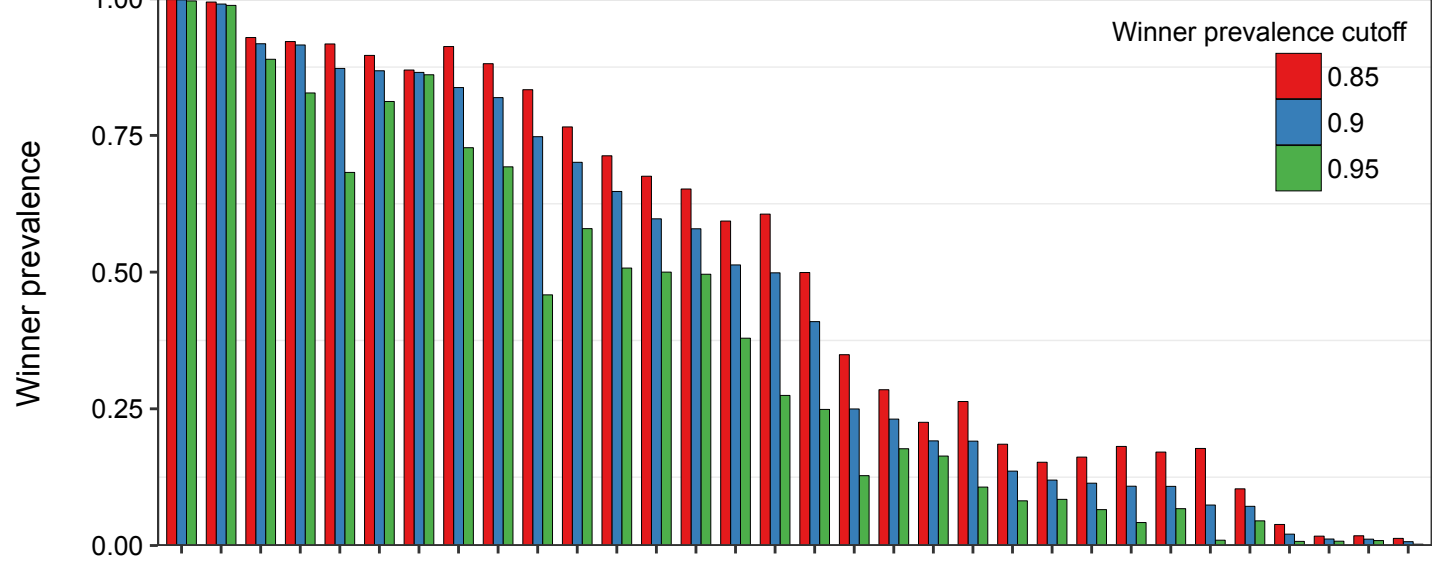

C

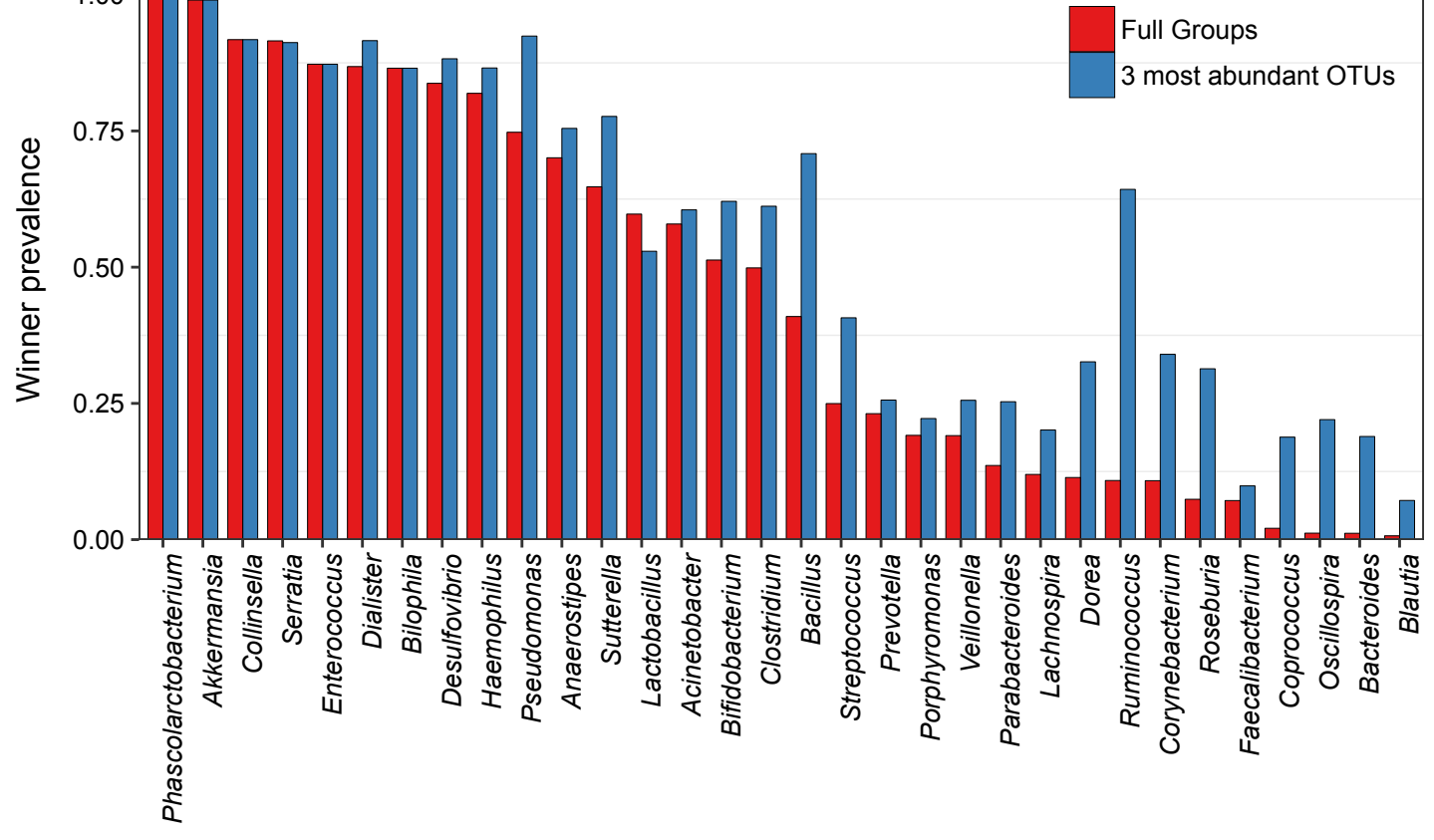

Supplement: Supplementary file 3 — Figure S2. Robustness of the winner prevalence estimate to different cutoffs. Bar plots show how the winner prevalence value changes for different genera if the cutoff was changed for OTU inclusion (A), for the lottery winner determination (B), and when only the three most abundant OTUs in each genera are considered (C). (PDF 33 kb) [file 40168_2018_571_MOESM3_ESM.pdf]

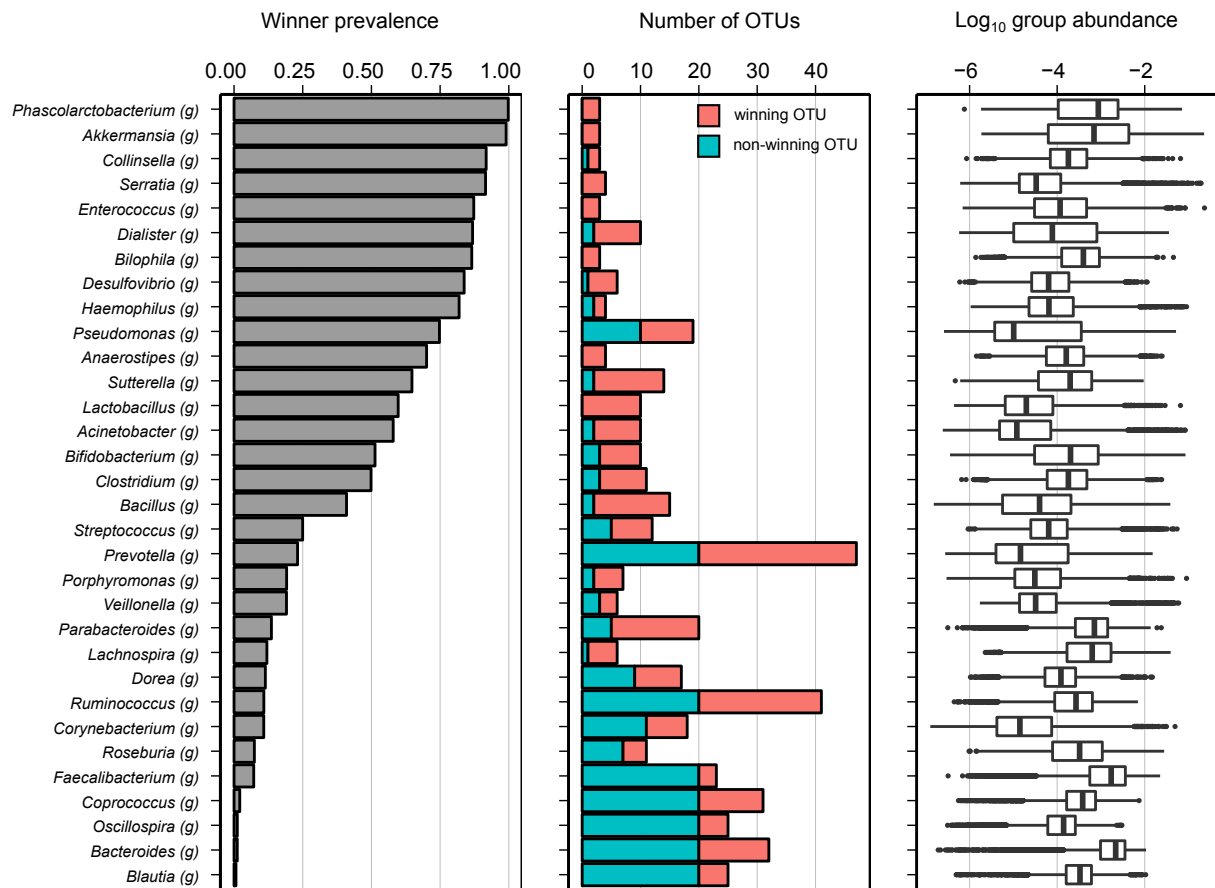

Supplement: Supplementary file 4 — Figure S3. Number of OTUs and abundance of the genera that were analyze in Fig. 1. The left panel illustrates the winner prevalence of each genus (as reported in Fig. 1). The middle panel illustrates the number of OTUs in each group (after filtration of rare OTUs; see the “Methods” section), as well as the number of OTUs that have been lottery winners (> 90% of the group abundance) in at least one sample. Finally, the right panel illustrates the distribution of the overall group abundance across all samples in the American Gut data. (PDF 274 kb) [file 40168_2018_571_MOESM4_ESM.pdf]

Within-group abundance

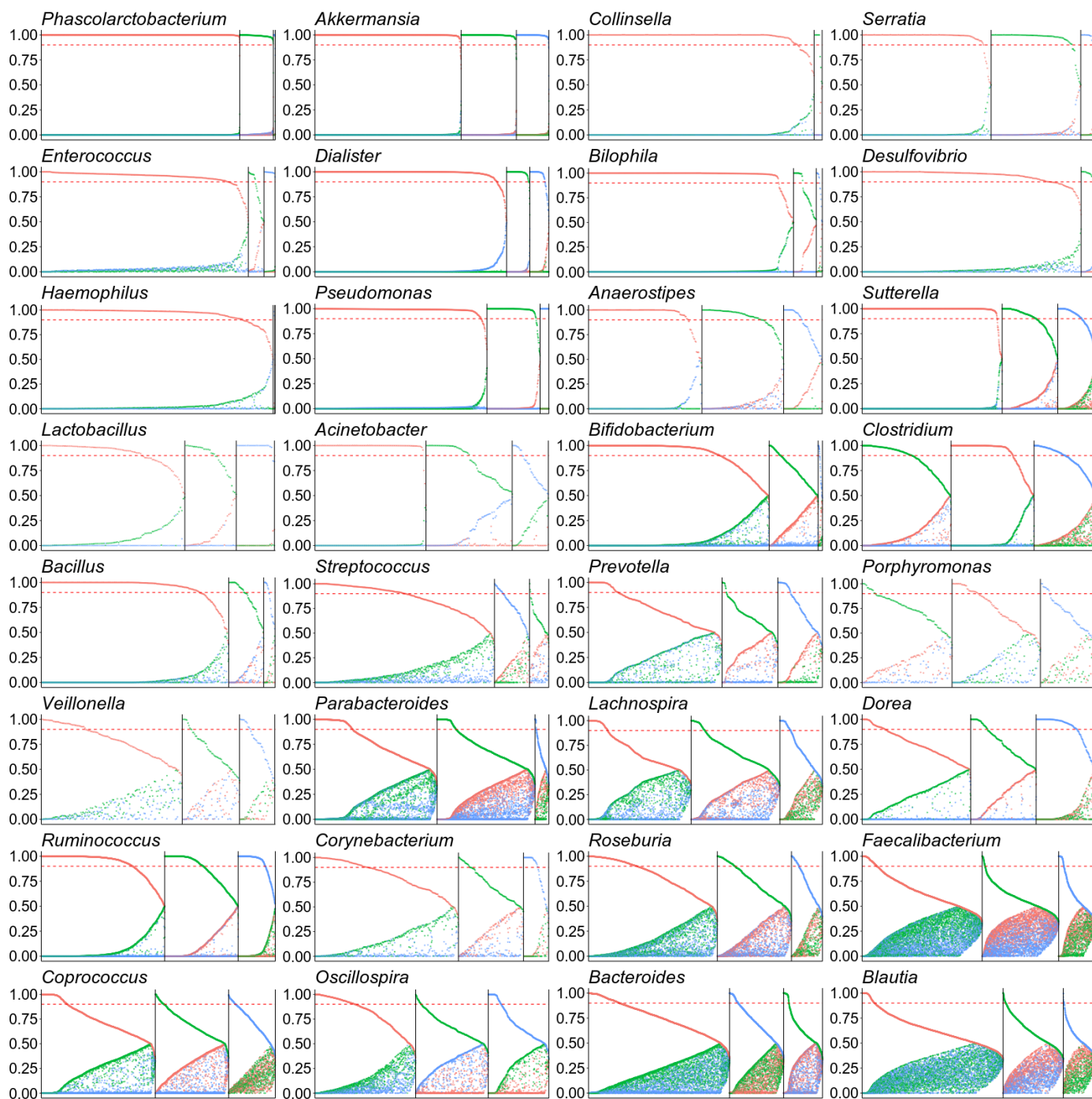

Microbiome samples

Supplement: Supplementary file 5 — Figure S4. Distribution of within-group abundances for all genera when considering only the three most abundant OTUs in each genus. Details are as in Fig. 3. (PDF 772 kb) [file 40168_2018_571_MOESM5_ESM.pdf]

Within-group abundance

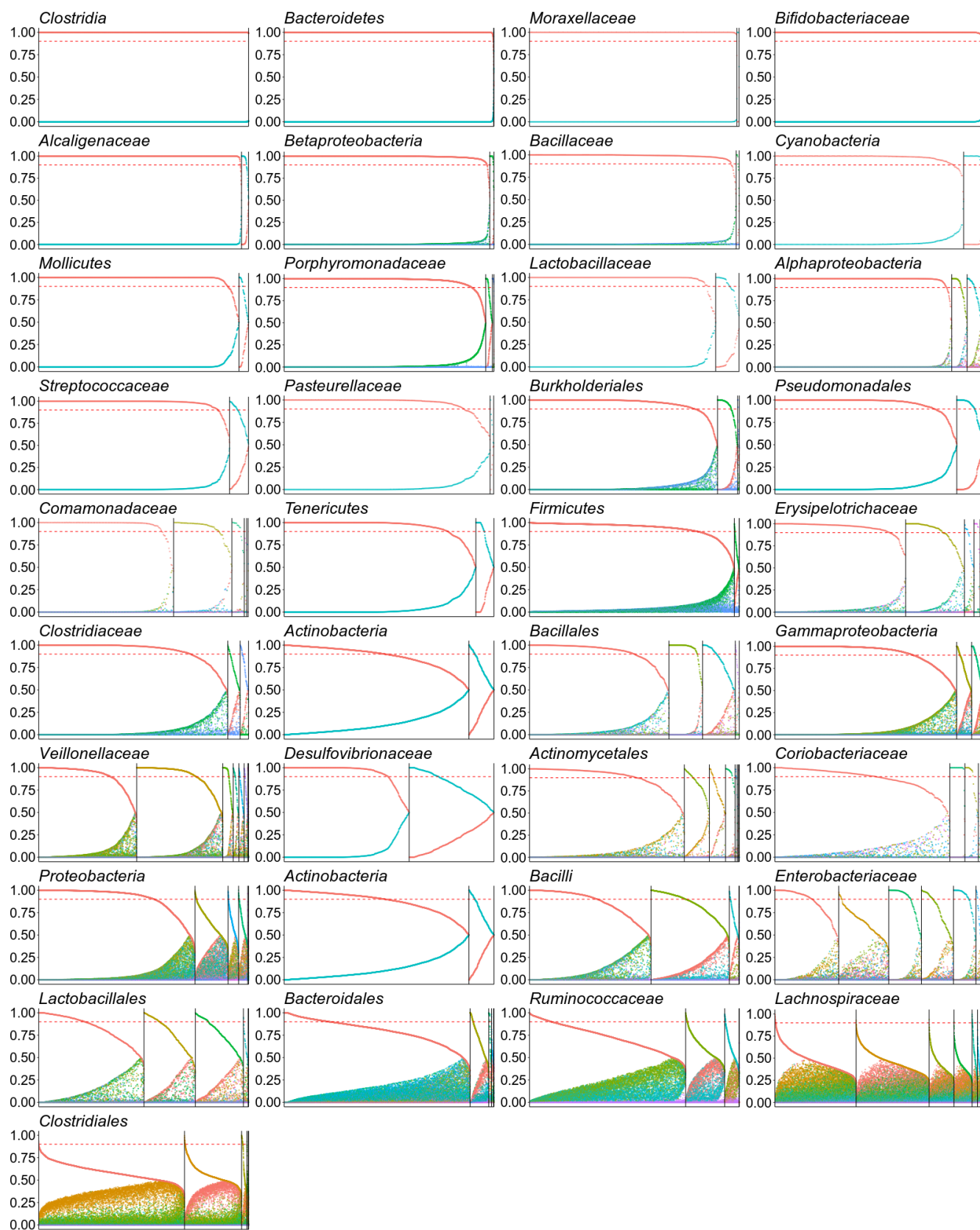

Microbiome samples

Supplement: Supplementary file 6 — Figure S5. Distribution of within-group abundances for higher-level taxonomic groups. Details are as in Fig. 4b, c. (PDF 813 kb) [file 40168_2018_571_MOESM6_ESM.pdf]

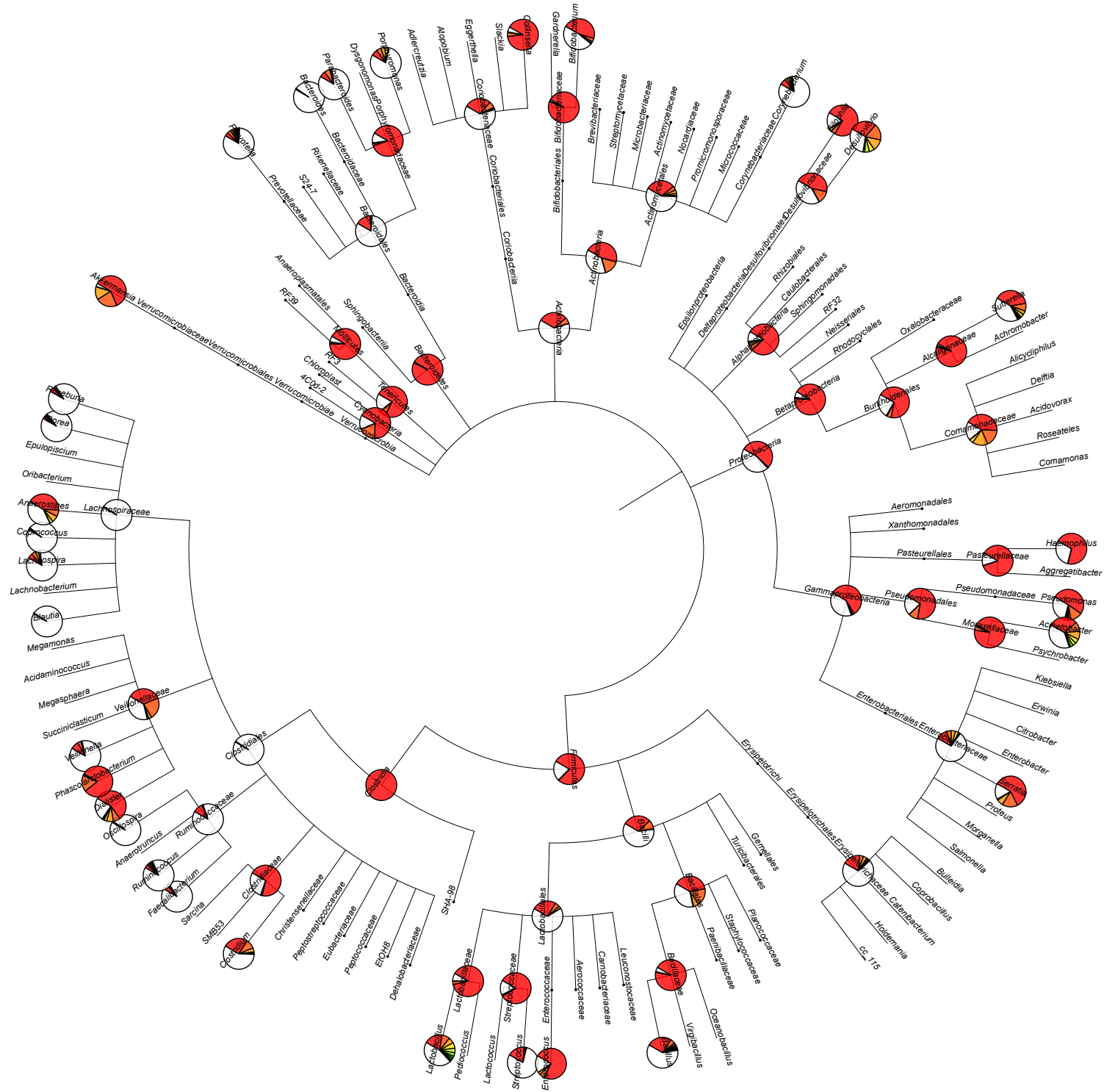

Supplement: Supplementary file 7 — Figure S6. A taxonomic tree, with assembly parameters displayed as pie charts. On each group that was analyzed in our study, we display the proportion of samples where different group members were lottery winners (> 90% abundance) using different colors. The proportion of samples without a winner is illustrated in white. With this visualization, the winner prevalence parameter is therefore denoted by the proportion of non-white pie chart, and the winner diversity parameter is proportional to the number and distribution of different colors in the pie chart. Groups without a pie chart were only used as subgroups to groups with pie charts. The tree was created with the interactive tree of life [72]. (PDF 73 kb) [file 40168_2018_571_MOESM7_ESM.pdf]

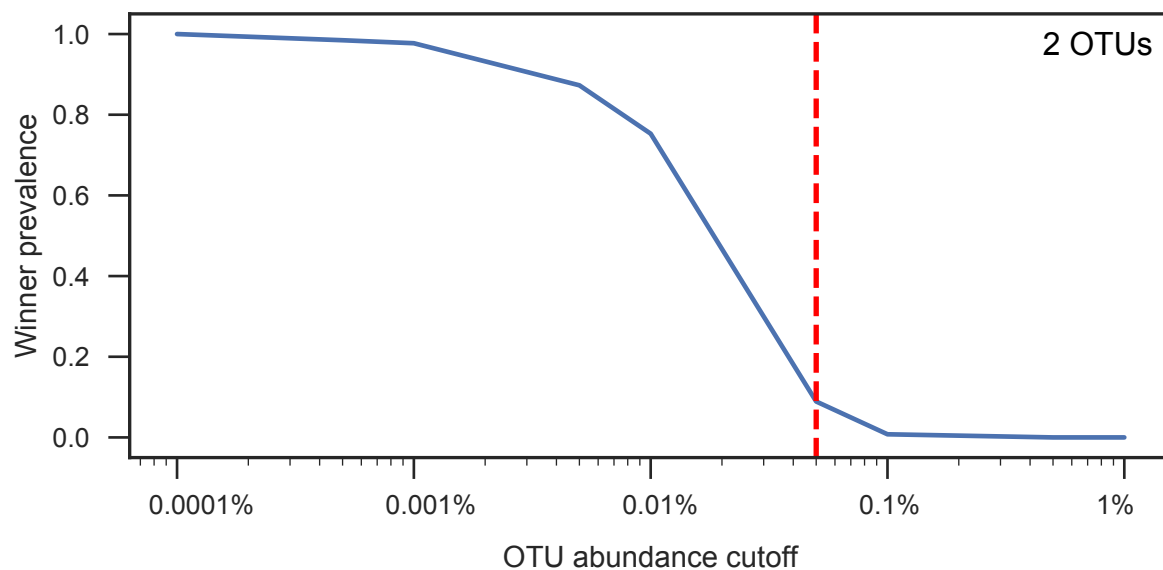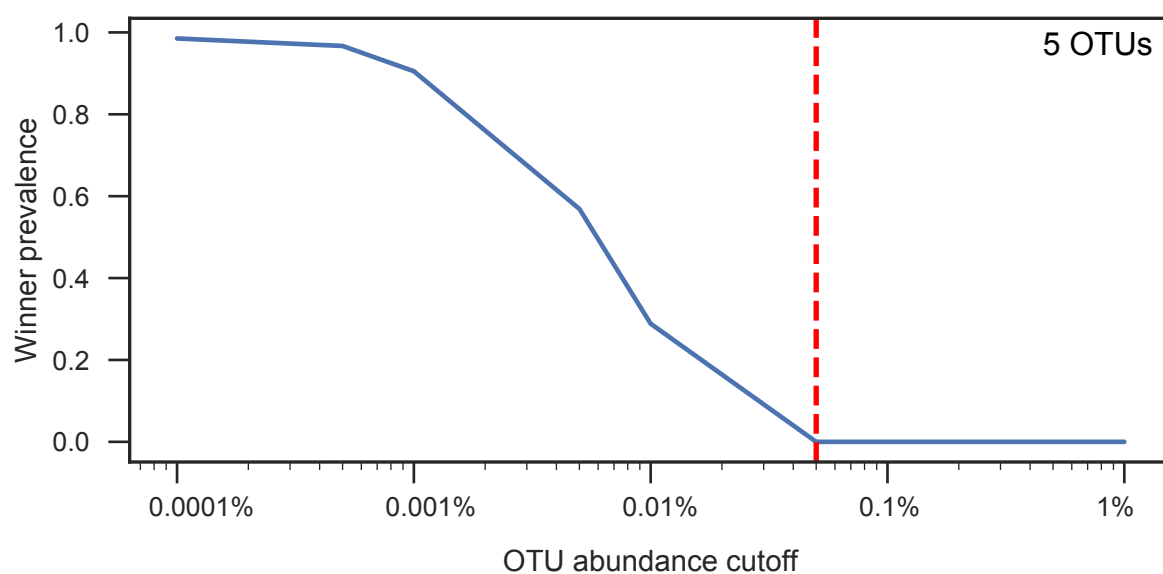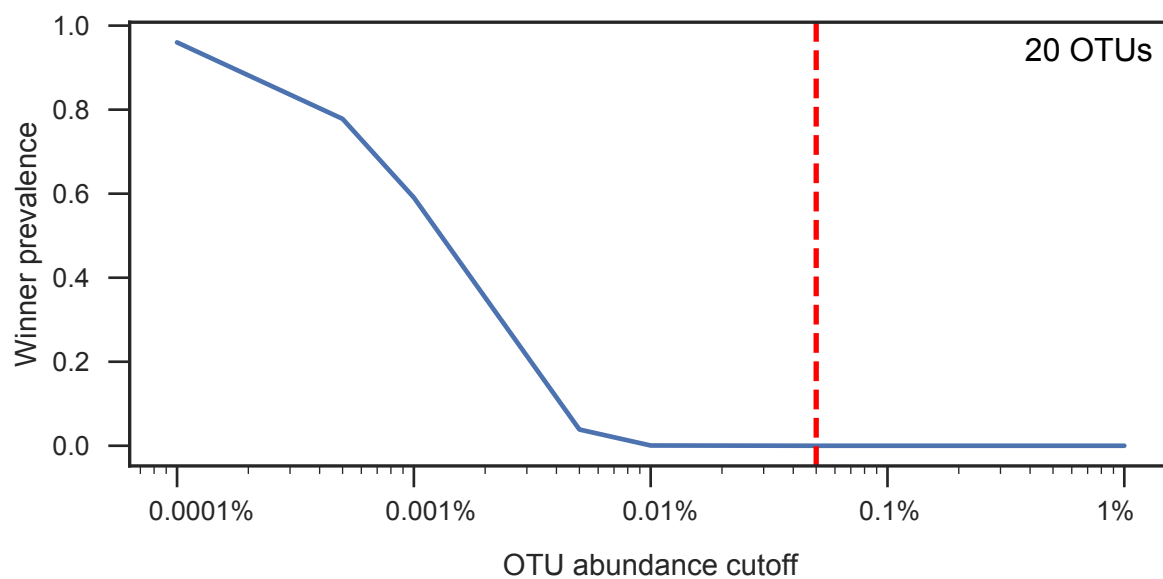

Supplement: Supplementary file 8 — Figure S7. Winner prevalence estimates in simulated groups at uniform abundance. Abundances have been simulated using a Poisson distribution assuming that OTUs are at a variable minimum abundance threshold (x-axis). If abundance estimates were perfect, we would expect a winner prevalence of zero, but noise associated with the sampling processes creates artificial winners. The dashed red line is the minimum abundance threshold used in our study. (PDF 18 kb) [file 40168_2018_571_MOESM8_ESM.pdf]
