# Supplementary material for: Competitive lottery-based assembly of selected clades in the human gut microbiome
Source: Microbiome. 2018 Oct 19;6:186. doi: 10.1186/s40168-018-0571-8 (PMC6195700; doi:10.1186/s40168-018-0571-8)
Supplement: Supplementary file 2 — Supporting text. (PDF 185 kb) [file 40168_2018_571_MOESM2_ESM.pdf]

Supporting Text

Null model for within group abundances

In order to determine a background expectation for the winner prevalence parameter, we used a stick breaking process to simulate the assembly of species under a null model in groups with varying number of members. Such a stick-breaking process is a commonly used model for the abundances of different species in ecology [1,2]. Briefly, a stick of unit length representing the group abundance is sequentially broken and the resulting pieces represent the abundance of each species in the group. Each sequential break in the stick is modelled with a Beta(1,1) distribution that divides the remaining group abundance into two pieces. Formally, the break points are given by:

$$\theta_k = \beta_k \prod_{i}^{k-1} (1 - \beta_i)$$

Where each β_k is drawn from a Beta(1,1) distribution and the abundance of each species is the length of the resulting segments between the breakpoints. Using this as our background distribution, we calculate the amount of the group abundance allocated to the most abundant member in 10,000 samples, and by examining the number of samples where this abundance exceeded 90% we assess an expected value of the winner prevalence parameter in this background distribution. We found that when for groups with 2 or 3 members the expected winner prevalence is ~20%, while for groups with 4 or more members the expected winner prevalence is ~10%. Given these probabilities the likelihood of observing winner prevalence >0.75 (which we used as a cutoff for defining lottery-like groups) with >1000 samples under this null model is < 1 / 20,000, and thus we were unlikely to erroneously identify lottery-like groups at these cutoffs.

Robustness Analysis of model and analysis parameters

Our characterization of the assembly of the gut microbiome depends on a number of cutoff values used in our analysis pipeline. To verify that our findings are not an artifact of the specific cutoff values used, we examined how our estimations of winner prevalence vary with these parameters and assessed whether our results change in a qualitative manner.

First, we examined whether our findings are robust to our OTU filtering procedure, and specifically, whether filtration of rare OTUs artificially increases the likelihood of observing lottery winners. In our main analysis we only included OTUs that appear at >0.05% abundance in at least 0.5% of samples (with a minimum of 10), to avoid noisy statistical estimation. We compared our findings with those obtained when filtering OTUs with abundance <0.01% and to those obtained without any filtering at all. We found that lowering the cutoff for OTU inclusion yielded only relatively minor changes in the calculated winner prevalence (Figure S2A), with the largest change observed in *Lactobacillus* where the winner prevalence dropped from 0.60 with our original cutoff to 0.52 with a cutoff of 0.01 and to 0.46 with no filtration whatsoever.

Second, we assessed how our results change as we vary the amount of the group's abundance allocation that is required to determine a lottery winner. In our main analysis we have defined lottery winners as OTUs that receive >90% of the group abundance (and see our justification for this cutoff above). We compare our findings (and specifically our calculation of the winner prevalence) to those obtained with either a looser cutoff (85%) or a stricter cutoff (95%). As expected, we found that at the looser cutoff, winner prevalence has increased, while at the stricter cutoff, winner prevalence has decreased (Figure S2B). However, in general, the shift in the winner prevalence was not substantial, with an absolute change of 0.05 in winner prevalence on average for the looser cutoff and an absolute change of 0.08 in winner prevalence on average for the stricter cutoff (Figure S2B). Moreover, the ordering of the various groups in terms of their winner prevalence did not markedly change ($\rho = 0.99$, $P < 5e-16$ at the looser cutoff and $\rho = 0.99$, $P < 5e-16$ at the stricter cutoff; Spearman correlation test). Furthermore, high winner prevalence groups such as *Akkermansia* and *Phascolarctobacterium* showed only a miniscule change in winner prevalence. The one group that shows the most extreme change in winner prevalence was *Pseudomonas*, which had a winner prevalence of 0.75 with our original cutoff and 0.46 with the stricter cutoff. This change could be attributed to the fact that in this group, the abundance allocated to the winner is generally between 90% and 95% (and see Figure 3)

Lastly, we examined how variation in the number of OTUs in each group affected the observed winner prevalence values. Specifically, we wanted to confirm that lottery groups are not simply an artifact of having a small number of OTUs in the groups, and accordingly having a higher likelihood of one of these OTUs reaching an abundance of >90% (and indeed, as demonstrated in Figure S3, there is a correlation between winner prevalence and the number of OTUs in the group). To test this, we repeated our analysis of winner prevalence but considered in each group only the three most abundant OTUs (renormalizing the abundance of these subsampled groups). As expected, this procedure generally increased the winner prevalence of the various groups, and more so for groups that were originally with low winner prevalence (and many OTUs; Figure S2C). For example, *Bacteroides* has 90 OTUs and a winner prevalence of 1%, but considering only the three most abundant OTUs from this group resulted in a winner prevalence of 19%. Importantly, however, even with this subsampling procedure, the separation between high and low winner prevalence group was generally preserved. Indeed, all of the 15 groups with winner prevalence >50% in our original analysis still exhibited >50% winner prevalence with this subsampling, and of the 17 groups with winner prevalence <50% in our original analysis, 14 still had winner prevalence <50% with that subsampling. Furthermore, in the subsampled groups the winner prevalence is still highly correlated to the full data ($\rho = 0.92$). Examining the distribution of species in each group using the original (Figure 3) and the subsampled groups (Figure S4), further demonstrate the clear difference between lottery-like groups like *Akkermansia* and non-lottery-like groups like *Blautia*. The one exception was *Ruminococcus* that had a relatively low winner prevalence in our original analysis (0.11) and a rather high winner prevalence when considering only the 3 most abundant OTUs (0.62), suggesting a complex assembly pattern whereby high abundance OTUs are able to exclude other high abundant OTUs but co-exist with many other low abundant OTUs.

Simulation analysis

In order to verify that low abundance OTUs were not artificially inflating our estimate of the winner prevalence due to statistical bias in estimating their abundance, we used a simulation study to determine whether our OTU filtering criterion were sufficiently high. We simulated a group of 8000 samples where the OTUs should be at equal abundance (*i.e.*, a non-lottery schema), assuming a range of different abundance cutoffs for OTU inclusion and a library size of 5000 reads (our minimum criterion for inclusion). The number of reads from each OTU was simulated using a Poisson distribution, OTU counts were transformed into relative abundances, and the winner prevalence was calculated in the same manner as done in our analysis above. We confirmed that including low abundance OTUs erroneously leads to higher winner prevalence values, but that using a cutoff of 0.05% for OTU inclusion results in low values of the winner prevalence parameter (Figure S7). Following this finding we used this cutoff for OTU inclusion in our analysis of the various datasets described in the main text.

References

1. MacArthur RH. On the relative abundance of bird species. Proc. Natl. Acad. Sci. U. S. A. 1957;43:293–5.

2. Higgins CL, Strauss RE. Modeling Stream Fish Assemblages with Niche Apportionment Models: Patterns, Processes, and Scale Dependence. Trans. Am. Fish. Soc. 2008;137:696–706.
